# Effect of hemodialysis and peritoneal dialysis on redox status in chronic renal failure patients: a comparative study

**DOI:** 10.1186/1476-511X-9-93

**Published:** 2010-09-03

**Authors:** Khedidja Mekki, Warda Taleb, Nassima Bouzidi, Abbou Kaddous, Malika Bouchenak

**Affiliations:** 1 Laboratoire de Nutrition Clinique et Métabolique, Département de Biologie, Faculté des Sciences, Université d'Oran 31100, Algérie; 2 Service de Néphrologie, Etablissement Hospitalo-Universitaire d'Oran, Algérie

## Abstract

**Objective:**

To investigate the effects of hemodialysis (HD) and periotoneal dialysis (PD) on oxidative stress in chronic renal failure patients (CRF).

**Methods:**

20 HD patients (M/F: 8/12, 36 ± 12 years) and 20 PD patients (M/F: 10/10, 40 ± 8 years) were compared with 20 end stage renal failure patients (CRF) (M/F: 4/16, 61 ± 13 years).

**Results:**

Thiobarbituric acid reactive substances (TBARS) values were elevated in HD and decreased in PD compared to CRF (P < 0.05). TBARS-VLDL and TBARS-HDL_2 _were decreased in HD and PD, compared to CRF (p < 0.05). TBARS-LDL were higher in HD compared to CRF (p < 0.05). No significant difference in TBARS-HDL_3 _values between the three groups. Carbonyls were increased in HD (p < 0.05) and PD (p < 0.01) compared to CRF. Plasma superoxide dismutase activity (SOD) was decreased in HD compared to CRF and PD (P < 0.05). Glutathion peroxidase activity (GSH-Px) was decreased in HD and PD (P < 0.005), compared to CRF. Decrease in catalase activity was noted only in PD compared to CRF (P < 0.05). An increase in nitric oxide was noted in HD compared to CRF (p < 0.05). Albumin concentrations were higher in HD and PD compared to CRF (P < 0.001). Whereas uric acid concentrations were decreased in HD (P < 0.001) compared to CRF and PD. Bilirubin values were similar in all groups. Increased values of iron were noted in HD and PD, compared to PD (p < 0.001).

**Conclusion:**

HD and PD aggravate oxidative stress generated by uremia. HD accentuates lipid and protein peroxidation, while PD aggravates protein oxidation. However, the activity of antioxidant enzymes was altered by both dialysis treatments.

## Introduction

Despite significant progress in dialysis technology, atherosclerosis and cardiovascular diseases (CVD) are the leading causes of morbidity and mortality in patients receiving renal replacement therapy (RRT). The estimated risk for cardiac events such as myocardial infarction is 3.5-50 times higher among patients requiring RRT than in the general population [1]. High rate of CVD morbidity and mortality in chronic renal failure (CRF) was attributed to a higher prevalence of both classical and nontraditional risk factors. Moreover, uremia itself contributes to cardiac pathology: many studies show that CRF is an independent risk factor for CV morbidity and mortality even after adjustment for traditional and nontraditional risk factors [2]. The classical risk factors for CVD (such as old age, hypertension, diabetes, smoking, dyslipidaemia, left ventricular hypertrophy, heart failure and physical inactivity) are over-represented in patients on RRT [2-4].

In recent years, oxidative stress (OS) has been postulated to be an important risk factor for cardiovascular disorders [5]. OS results from an imbalance between oxidant production and antioxidant defence mechanisms with increased levels of pro-oxidants leading to tissue damage [6]. Antioxidants can be divided into intracellular and extracellular antioxidants. Intracellular enzymatic antioxidants are superoxide dismutase (SOD), catalase, and glutathione peroxidase, which convert substrates (superoxide anion radicals and hydrogen peroxide) to less reactive forms. Several extracellular antioxidants, such as albumin, bilirubin and urate, prevent free radical reaction by sequestering transition metal ions by chelation in plasma [6].

Several studies have demonstrated increased oxidative stress in patients with CRF, including accumulation of reactive carbonyl compounds as markers of elevated protein peroxidation [5,7,8], increased concentrations of thiobarbituric acid-reactive substances and malondialdehyde in plasma as markers of high lipid peroxidation [9-11].

One reason for OS in patients with renal failure is the underlying disease itself. Renal toxicity and immunological disorders of the kidney result in an elevated formation of reactive oxygen species (ROS) active in the pathogenesis of kidney disease. However, treatment procedures were also shown to induce OS. Oxidative stress is particularly detrimental in patients receiving hemodialysis (HD) because there is massive and repeated at each dialysis session due to the contact of blood with dialysis membranes, facing to a chronic deficit in antioxidant defense system (8). Moreover, Several lines of evidence have indicated that oxidative metabolism in peripheral and peritoneal phagocytes is activated during peritoneal dialysis (PD) with conventional dialysate characterized by high concentration of glucose, by glucose degradation products (GDP), and by low pH and high osmolality [12]. Bioincompatibility of PD solutions seems to play a central role in the increase of ROS production [13].

Moreover, It had been hypothesized that a deficiency of vascular nitric oxide (NO) might be involved in the accelerated atherosclerosis and dramatic cardiovascular mortality observed in patients with CRF [14]. Endothelial dysfunction defined as the impaired ability of vascular endothelium to stimulate vasodilation, plays a key role in the development of atherosclerosis in CRF. The major cause of the endothelial dysfunction is decreased bioavailability of NO, a potent biological vasodilator produced in vascular endothelium from L-arginine by the endothelial NO synthase (eNOS). Another important role of endothelial NO is the protection of the vascular wall from the OS induced by its own metabolic products and by the oxidation products of lipids and lipoproteins [15].

Thus, this study was carried out in order to investigate the effects of hemodialysis (HD) and periotoneal dialysis (PD) on lipid peroxidation and protein oxidation and antioxidant defence in patients with chronic renal failure (CRF).

## Subjects and Methods

The study was carried out on 60 CRF patients. They included 20 end-stage renal failure patients (CRF), 20 HD patients and 20 PD patients (Table 1). In the selection of the CRF patients, we excluded all those with systemic disease such as diabetes, liver disorders and those affected by nephrotic syndrome. None of the patients were taking lipid-lowering drugs or antioxydant supplements. The drugs they were using included: calcium channel blockers, vit D, calcium carbonate and erythropoietin. CRF patients included 20 subjects who had a mean value of creatinine clearance of 9.2 ± 2.1 ml/min (range 5-13) as calculated from plasma creatinine according to Cockroft and Gault formula [16] [GFR = (140-age) × body weight × 1.23/creatinine. In women, this value was multiplied by 0.85]. Dietary protein and phosphate intake were restricted in these patients. HD patients were on standard bicarbonate HD using polysulfone membrane. Patients were dialyzed since 12 to 60 months, three times a week, each session lasting 4 h. PD patients were in dialysis since 3 to 48 months, using a standard procedure (four exchanges: three isotonic 1.36% glucose solutions, then a hypertonic one at 3.86% glucose).

**Table 1:** Clinical and biochemical characteristics of the patients

	CRF	HD	PD
Patients	N = 20	N = 20	N = 20

Age (years)	61 ± 13	36 ± 12	40 ± 8

Weight (kg)	71.5 ± 8.07	56.71 ± 13.87	65.87 ± 18.22

BMI (Kg/m^2^)	27 ± 3	21 ± 4	23 ± 5

Sex ratio (M/F)	4/16	8/12	10/10

Dialysis duration (months)	-	12 - 60	3 - 48

Glucose (g. L^-1^)	0.90 ± 0.06	0.85 ± 0.02	0.95 ± 0.06

Creatinin (μmol.ml^-1^)	221 ± 94	834 ± 47	735 ± 194

Urea (mmol.L^-1^)	12 ± 4	14 ± 1	12 ± 0.8

Total proteins (g. L^-1^)	66 ± 0.6	60 ± 1.2	57 ± 0.9

Data are expressed in mean ± SD. BMI: Body mass index (weight kg/height m^2^). SBP: Systolic blood pressure, DBP: Diastolic blood pressure.

All patients were treated at the Nephrology ward of the University Hospital of Oran. The purpose of this study was explained to the subjects and the investigation was carried out with their consent. The experimental protocol was approved by the Committee for Research on Human Subjects of Oran.

### Assays

In all patients, blood samples were drawn after a 12-hours overnight fast by antecubital venipuncture in CRF and PD patients and by the dialysis fistule in HD patients. Samples were collected by low speed centrifugation at 3000 × g at 5°C, for 15 min, and were preserved with 0.1% Na2 EDTA and 0.02% sodium azide.

#### Lipids, proteins and apolipoproteins analysis

Triacylglycerols (TG) and total cholesterol (TC) were determined by colorimetric methods (*BioMérieux *Kits, *France*). Plasma high density lipoprotein-cholesterol (HDL-C) was determined enzymatically using the CHOD-PAP kit after precipitation of the chylomicrons, very low density lipoprotein cholesterol and Low density lipoprotein cholesterol (LDL-C) with phosphotungstic acid and Mg^++ ^*(BioMérieux Kits, SA-France)*. Plasma LDL-C was determined enzymatically using the CHOD-PAP kit after precipitation of LDL. Plasma apolipoproteins (apo) A-I and B were measured by immunoturbidimetric method (*Human kit, Germany)*.

#### Isolation of plasma VLDL-LDL-HDL_2 _and HDL_3_

Lipoproteins were isolated by precipitation using MgCl2 and phosphotungstate (Sigma Chemical Company, France) by the method of Burstein *et al*., 1970 [17]. HDL_2 _and HDL_3 _were separated by precipitation according to the method of Burstein *et al*., 1989 [18] using MgCl2 and dextran sulfate weight 500,000 (Sigma Chemical Company, France).

#### Lipid and protein peroxidation

Lipid peroxidation was estimated by measuring thiobarbituric acid reactive substances (TBARS) concentrations according to the method of Quintanilha *et al *[19], using tetramethoxypropane (Prolabo) as precursor of malondialdehyde. TBARS was analyzed in plasma and in each lipoprotein. One milliliter of diluted sample (protein concentration about 2 mg/ml) was added to 2 ml of thiobarbituric acid (final concentration, 0.017 mmol/L), butylated hydroxytoluene (concentration, 3.36 mmol/L) and incubated for 15 min at 100°C. After cooling and centrifugation, the absorbance of supernatant was measured at 535 nm. Data were expressed as mmol of TBARS produced/ml of plasma.

Oxidized proteins were estimated by measuring carbonyls concentrations according to the method of Levine *et al *[20] using the 2.4-dinitrophenylhydrazine (DNPH).

#### Antioxidant measurements

Superoxide dismutase (SOD; EC 1.15.1.1) and Glutathione peroxidase (GSH-Px; EC 1.11.1.9) were determined by Cayman Chemical kit. SOD activity was assessed at 440 nm by measuring the dismutation of superoxide radicals generated by xanthine oxidase and hypoxanthine. GSH-Px activity was measured indirectly by a coupled reaction with glutathione reductase (G-Red). Oxidized glutathione (GSSG), produced upon reduction of an organic hydroperoxide by GSH-Px was recycled to its reduced state by G-Red and NADPH. The oxidation of NADPH to NADP+ is accompanied by a decrease in absorbance at 340 nm. Catalase (CAT; EC 1.11.1.6) activity was measured at 25°C using the Aebi method [21] by measuring the rate of decomposition of H_2_O_2 _at 240 nm. Colorimetric methods were used for the determination of albumin, urate (Kits Boehringer, Mannheim, Germany), iron and bilirubin (Biolabo kit, France). C-Reactive Protein (CRP) was measured by the immunoturbidimetric method (Fumouze, France).

#### Determination of plasma nitric oxide (N0)

NO determination was performed using the Griess reagent (sulfamide and n-naphtyl-ethylene diamine) (Cortas & Wakid., 1990) [22] method. Plasma was clarified by zinc sulfate sodium and NO_3 _was then reduced to NO_2 _by cadmium overnight at 20°C under shaking. Samples were added to the Griess reagent and incubated for 20 min at room temperature. Absorbance was measured at 540 nm. Sodium nitrite was used for a standard curve.

### Statistical analysis

Statistical analysis was performed using STATISTICA 6.0 (for windows, StatSoft Inc. software, Tulsa, OK, USA). Data are presented as mean ± standard deviation (SD). Data normality and the distribution of the variables were tested by the Kolmogorov-Smirnov test. The difference between the means from the different groups was checked by ANOVA adjusted for multiple comparisons. Depending on the normality of distribution of variables, the comparisons between groups were performed using the unpaired Student's t-test, 1-way analysis of variance (ANOVA) or the Mann-Whitney U-test when results were nonparametrically distributed. *P *< 0.05 was considered statistically significant.

## Results

### Lipids and apolipoproteins parameters (Table 2)

**Table 2:** Lipids, apolipoproteins and atherogenic indices of the study groups

	CRF	HD	PD
TG (mmol.L^-1^)	3.11 ± 0.4	1.48 ± 0.62**	1.7 ± 0.42**

TC (mmol.L^-1^)	6.02 ± 2.48	6.82 ± 1.29	4.7 ± 1.57 #

HDL-C (mmol.L^-1^)	1.29 ± 0.94	1.09 ± 0.41	1.07 ± 0.53

LDL-C (mmol.L^-1^)	2.25 ± 0.62	3.76 ± 0.27**#	2.35 ± 0.40

Apo A-I (g. L^-1^)	1.07 ± 0.53	1.11 ± 0.14	0.89 ± 0.48

Apo B (g. L^-1^)	0.34 ± 0.05	0.27 ± 0.07	0.30 ± 0.04

TC/HDL-C	7.38 ± 0.34	4.88 ± 0.73 *	2.27 ± 0.48**###

LDL-C/HDL-C	2.50 ± 0.94	2.96 ± 0.35	2.59 ± 1.36

Apo AI/Apo B	2.43 ± 0.85	3.89 ± 0.67	3.04 ± 1.75

CRF: End stage chronic renal failure patients. HD: patients on hemodialysis. PD: Patients on peritoneal dialysis. Data are presented as the mean ± SD. HD group and PD group *vs *CRF group # HD group vs PD group. *# (P < 0.05); **##(P < 0.01); ###(P < 0.001)

A significant decrease in TG values was noted in HD (-52%) and PD (-45%) compared to CRF (p < 0.01), while no significant difference was noted. No significant difference was noted in TC values in HD and PD groups compared to CRF group. However, a significant decrease in TC values by 31% was noted in PD compared to HD (p < 0.05).

A significant increase by 40% in LDL-C values was observed in HD compared to CRF (p < 0.01). Values were similar in PD and CRF. However, LDL-C concentrations were elevated by 37% in HD compared to PD (p < 0.05).

Similar values of apo A-I, apo B, HDL-C concentrations, LDL-C/HDL-C and Apo AI/Apo B ratios were noted in HD, PD and CRF groups. However, values of TC/HDL-C ratio were decreased by 34% and 69%, respectively in HD (p < 0.05) and PD (p < 0.01), compared to CRF. Moreover, values of this ratio were diminished by 53% in PD compared to HD (p < 0.001).

### Oxidative status (Table 3)

**Table 3:** Oxidative status in the study groups

	CRF	HD	PD
TBARS (μmol.L^-1^)	0.12 ± 0.01	0.23 ± 0.02*	0.18 ± 0.01*

TBARS-VLDL (μmol.L^-1^)	0.55 ± 0.02	0.10 ± 0.05**	0.026 ± 0.014**

TBARS-LDL (μmol.mL^1^)	0.038 ± 0.002	0.055 ± 0.023*	0.052 ± 0.014*

TBARS-HDL_2 _(μmol.mL^1^)	0.09 ± 0.01	0.047 ± 0.008*	0.023 ± 0.01*#

TBARS-HDL_3 _(μmol.mL^1^)	0.196 ± 0.05	0.105 ± 0.070	0.231 ± 0.049

Carbonyls (μmol.mL^1^)	0.3 ± 0.16	0.92 ± 0.15*	1.90 ± 0.10**##

CRF: End stage chronic renal failure patients. HD: patients on hemodialysis. PD: Patients on peritoneal dialysis. Data are presented as the mean ± SD. * HD group and PD group *vs *CRF group # HD group vs PD group. *#(P < 0.05); **##(P < 0.01)

Compared to CRF, TBARS values were elevated in HD (P < 0.05) and PD (p < 0.05). A significant decrease in TBARS-VLDL by 82% and 95% was noted in HD and PD groups, respectively, compared to CRF group (p < 0.01). TBARS-LDL were significantly higher in HD and PD groups compared to CRF group (p < 0.05). There was no significant difference in TBARS-HDL_3 _values between the three groups. TBARS-HDL_2 _values were decreased by 48% and 74% in HD and PD, respectively, compared to CRF (p < 0.05). Values were more lower by 51% in PD compared to HD (p < 0.05).

Carbonyls values were significantly increased by 67% in HD (p < 0.05) and by 84% in PD (p < 0.01) compared to CRF. Carbonyls concentrations were more elevated (+52%) in PD than in HD (p < 0.01).

### Antioxidative status (Table 4)

**Table 4:** Antioxidative status in the study groups

	CRF	HD	PD
SOD (U.ml^-1^)	82.10 ± 1.53	70.08 ± 4.20*	80.12 ± 0.38#

GSH-Px (U.ml^-1^)	5.92 ± 0.53	4.12 ± 0.3*	4.08 ± 0.06*

Catalase (U.ml^-1^)	85.45 ± 4.74	80.18 ± 1.32	65.78 ± 3.45**#

Nitric oxide (μmol.L^-1^)	22.53 ± 7.24	30.57 ± 8.7*	20.52 ± 1.58

Albumins (g.L^-1^)	32.95 ± 0.25	30.78 ± 1.18*	29.50 ± 0.07*

Urate (μmol/L)	513.94 ± 171.61	382.09 ± 66.07***	349.63 ± 167.2***

Bilirubin (μmol/L)	6.29 ± 2.60	5.01 ± 0.60	8.39 ± 6.76

Iron (μmol/L)	33.08 ± 7.71	55.17 ± 1.53 ***	40.59 ± 3.07###

CRP (mg L^-1^)	6.5 ± 0.2	8.2 ± 0.2*	8.8 ± 0.8*

CRF: End stage chronic renal failure patients. HD: patients on hemodialysis. PD: Patients on peritoneal dialysis. Data are presented as the mean ± SD. * HD group and PD group *vs *CRF group # HD group vs PD group. *# (P < 0.05); **(P < 0.01); ***###(P < 0.001)

Plasma SOD activity was decreased in HD compared to CRF (-15%; P < 0.05) and PD (-13%; P < 0.05). Similar SOD activity was noted in PD and CRF. GSH-Px activity was decreased by 23% in HD and PD (P < 0.005), compared to CRF. Decrease in catalase activity was noted only in PD compared to CRF (-22%; P < 0.05) and HD (-18%; P < 0.05). A significant increase by 26% in NO concentrations was noted in HD compared to CRF (p < 0.05).

Albumin concentrations were diminished in HD and PD compared to CRF (P < 0.05). Uric acid concentrations were decreased in HD and PD (P < 0.001) compared to CRF. Bilirubin values were similar in the three groups. Whereas, an increase by 67% and 59% in iron values was noted in HD and PD, compared to PD (p < 0.001). C-Reactive Protein concentrations were higher in HD and PD groups compared to CRF (p < 0.05).

## Discussion

A comparative study was conducted in CRF patients, in order to evaluate the effect of HD and PD on lipid peroxidation, protein oxidation and antioxidant defence. The case-control studies agree that CRF patients have increased OS produced by an imbalance between pro- and anti-oxidant capacities [23-25]. This oxidative stress is responsible for the peroxidation of macromolecules such as lipids and proteins causing significant damage. Several pathophysiologic explanations have been claimed; some attribute it to malnutrition and hypoalbuminemia having in theses cases low availability of «thiol»; others to «uremic status» itself with solute retention that may favor their pathogenicity; and others to the association of comorbid factors such as advanced age, diabetes, and inflammatory and infectious phenomena [26,27].

Dyslipidemia is one of the main risk factors of cardiovascular complications in patients with CRF. In our study, patients on HD and PD have lower level of plasma triglycerides, than undialyzed CRF patients. Total cholesterol, HDL-C, apo A-I and B were unchanged according to dialysis treatment. However, HD causes an elevation of LDL-C. These results were different from our previous investigations in which we established more elevated TG values in HD patients treated by a cuprophan membrane [28].

The present study demonstrates that HD and PD cause an increase in oxidative stress. HD and PD leads to a lipid and protein peroxidation. OS was also affirmed by the decrease in the activity of antioxidant enzymes. Peroxidations of LDL have been postulated as important factors in the development of atherosclerosis. Increased levels of lipid peroxides are detected in patients with atherosclerotic disease. Also, the susceptibility of LDL to oxidation in vitro has been associated with the severity of coronary atheroselerosis in patients with CVD [29].

The formation of carbonyls is an early marker of protein oxidation. Carbonyls stress in uremia was related to decreased removal of chemically modified proteins by glomerular filtration. Reactive carbonyl compounds formed by the oxidation of carbohydrates and lipids may indirectly lead to advanced glycation or lipoxidation proteins, contributing to the long-term complications associated with CRF [30].

Free radicals are the source of lipid peroxidation derived from oxygen, and the first line of defense against them is SOD. Its function is to catalyse the conversion of superoxide radicals to hydrogen peroxide (H_2_O_2_). Hence, the decreased SOD activity in HD suggests that the accumulation of superoxide anion radical might be responsible for increased lipid peroxidation [6]. Moreover, GSH-Px is responsible for most of the decomposition of lipid peroxide and may thus protect the cell from the deleterious effects of peroxides. H_2_O_2 _in the presence of sufficient catalase activity will be converted to harmless H_2_O and O_2_. In the present investigation, lower GSH-Px activity was observed in HD and PD. GSH-Px is an enzyme that scavenges H_2_O_2 _and lipid peroxides [6]. Our result explained the increase in TBARS levels. In this study, NO concentrations were elevated in HD patients however PD seems to not alter their levels. The important role of endothelial NO is the protection of the vascular wall from the OS induced by its own metabolic products and by the oxidation products of lipids and lipoproteins [14,15].

Bilirubin, uric acid and plasma albumin concentrations are the primary defense against OS in extracellular fluids results [30,31], generated during normal metabolism or introduced in the body by the consumption of dietary products rich in antioxidants. Knowing that our HD patients had a food intake characterized by reduced fruit and vegetables intake, this unbalanced diet contributes to a significant oxidative stress. Antioxidants are essentially made by fruit and vegetables that are rich in vitamins (A, C, E), trace - elements and other polyphenols.

The determination of plasma iron provides excellent guidance on the OS status of patients. Iron values were elevated in HD and PD patients. Iron plays a crucial role in the initiation and propagation of radical reactions allowing the formation of hydroxyl radicals (OH°). Iron also catalyzes the decomposition of lipid peroxides (ROOH) by transforming them into alkoxyl radicals (RO°) and peroxyl (ROO°) that amplify the process of lipid peroxidation. However, iron increase the generation of oxygen free radicals, leading to the formation of lipid peroxides [7]. In our study, CRP values were greater than 5 mg.L^-1 ^in the CRF group and values were more elevated in HD and PD. CRP is a prominent product of the inflammatory response syndrome and a marker of overall and cardiovascular death in the general population as well as in CRF patients [32]. The view has been brought forward that a chronic inflammatory response may be the primary cause of increased oxidative stress in CRF patients [33]. However, it could also be vice versa. The imbalance between free radical formation and neutralization in dialysis patients may be the causative factor for the activation of an inflammatory cascade by a variety of potential stimulators in uremia and dialysis. A common signaling occurs via generation of oxygen-free radicals, activation of the transcription factor nuclear factor-kappa B (NF-B) and induction of a number of genes such as adhesion molecules, cytokines, and chemokines. The result may be IL-6 stimulated production of CRP by the liver [32].

However, the problem to be addressed is to know why HD and PD aggravate oxidative stress of uremic patients. HD further worsens this condition mainly by losses of hydrophilic unbound small molecular weight substances such as vitamin C, trace elements and enzyme regulatory compounds. Moreover, inflammatory state plays a critical role in the production of oxidants contributing further to aggravate the pro-oxidant status of uremic patients [7,8,30,31]. Dialyser interactions and the microbial contamination or pyrogen content of the dialysate, the possible prooxidant effect of a number of metabolites, found at high concentrations in the patients' plasma, including uric acid have been suggested as the three major causes of OS. In the HD population, as the interaction between dialysis membranes and blood neutrophils can trigger the release of oxygen-free radicals and oxidizing agents, such as superoxide anion, hydrogen peroxide and myeloperoxidase. In turn, these molecules contribute to the oxidation of lipid by products, proteins and nucleic acids. This oxidation has several pathophysiological consequences, including enhanced atherogenicity of Ox-LDL, as well as accelerated demise of circulating erythrocytes, leading to a shorter life span [34,35].

Infections occurring in peritoneal dialysis patients are more frequent than in general population. Risk factors in those patients are multiple such as immune deficit and cutaneous port of entry. Our patients were treated by a conventional peritoneal dialysis considered as bioincompatible. Repeated and long-term exposure to conventional glucose-based peritoneal dialysis fluids plays a central role in the pathogenesis of the functional and structural changes of the peritoneal membrane. Low pH, high glucose concentration and heat sterilization represent major factors of low biocompatibility [31]. GDP are formed during heat sterilization (glycoxidation) and storage. GDP can bind protein and form AGEs (Advanced Glycation End-products), which can also result from the binding of glucose to free NH_2 _residues of proteins (glycation).

In conclusion, this study demonstrates that hemodialysis and peritoneal dialysis exacerbate oxidative stress generated by uremia. HD and PD accentuate lipid and protein peroxidation, moreover, the activity of antioxidant enzymes was altered by both dialysis treatment

## Competing interests

The authors declare that they have no competing interests.

## Authors' contributions

KM: Study design, statistical analysis, results interpretation, literature search and drafting of the manuscript. WT: Data collection and biochemical analysis. NB: Biochemical analysis. AK: Data collection. MB: Manuscript preparation. All authors reviewed and approved the final version of the paper and approved of its submission to Lipids in Health and Disease.
